# Report of the Committee on Mechanical Dentistry, to Southern Dental Association, 1874

**Published:** 1874-11

**Authors:** 


### ARTICLE II.

### *Report of the Committee on Mechanical Dentistry,*

To the Southern Dental Association, 1874.

No very striking or important invention has been developed in Mechanical Dentistry during the past year. But while so many advances are being instituted and startling developments made in the operative department, your Committee does not think that the fact of their having nothing specially new to report, should excite concern. Not that the question has lost its interest, or that Mechanical Dentistry is losing its importance, but it is thought that what we most need is not something more available, so much as that a better use be made of what we are already possessed of. The eager cry, etc., restless search for the new and novel, for something of easy manipulation is to be deprecated as injurious to that patient culture from which alone can result those productions of art which are at once our admiration and our despair.

Good, honest, faithful work is the great necessity of the hour. What the profession most needs at this time, is men who are not afraid to extend the same amount of labor of body and brain on replacement that they are willing to give to the natural organs. Banished as this department of our practice is to an out of the way room, and given in charge of a subordinate whose only ideas on such subjects are such as he has picked up from hurried instructions, imparted while handing him an impression and shade tooth ; it is not to be wondered at, that our work in this line has fallen in our own esteem, and is slightly valued by those for whom it is executed ?

We should endeavor to restore the Laboratory to something of its former dignity ; its full and original importance it may never have, and it is well perhaps that it is so. The advances of art in Operative Dentistry, forbid the wholesale loss of teeth that formerly occurred. But in the effort at accomplishing so much in the way of saving the natural organs, have we not lost sight of the many for whom we have extended our best efforts ? We have only postponed the evil day.

The manufacturers of teeth evidently have not forgotten these, but have kept pace with the demands of the times. But is it fair for us to be led by those outside of our profession ? Is it not a reflection on ourselves that men who have no such lively interest in this question as we, should be the suggestors of ideas and forms of beauty, such as we only idly dream of ?

No ; we want honest workers in the Laboratory. The reaction which we have so sadly needed has, we think, evidently begun, and good solid gold and other substantial metallic plates are reasserting their right to a place in the mouth, while the cheap and horrid vulcanite is passing away. Having had its day, it is time ! Emblem in worth of the knavery which has fattened on it and of the stupidity and ignorance which has manipulated it, it is to be hoped that this base is at least taking its proper place in the list of materials for dental plates—at the bottom.

But while this reaction is going on, it were well if it could extend still further. This whole curse of cheap work has only to be ventilated to show its hollowness and want of truth. The dentist who bargains for gold plate fit to wear and makes it half alloy, needs converting to a purer faith, a holier practice. The man who allows himself to be persuaded into buying cheap teeth because they are just as good if not better than those that cost three times as much, (or at least the sellers say so ;) the one who will persuade his patient into wearing a rubber set, saying it is just as good if not better than gold, etc., all this to save a few cents and and a little trouble, or perhaps to save the mortification of acknowledging that he does not know how to work gold—such need converting.

Cannot the "best men in the profession," who "have been driven from the Laboratory in disgust," come forth and show that there is a better way than this present and past? Their patients heed them on other subjects, and are wary of the men who will fill their teeth at half price,—will they not heed them also in this?

Your Committee would repeat that the need of the hour is better men in the Laboratory. The times demand it, our patients need it, and we should not be slow to show them that they do. So long as there are hundreds who never made any other than a rubber plate, and, while we may count on our fingers almost the good plate workers, it is surely time for reform. We talk of educating our patients in other things, we carefully instil into their minds the superiority of gold over amalgam,—the same teaching in this department, as carefully performed, should produce gratifying results.

Of the rubber litigation, etc., its results your Committee deem it scarcely worth while to comment on, except in so far as it furnishes additional arguments in favor of a higher standard of work than at present exists. If this controversy shall result in compelling the more general disuse of vulcanite, (a result by no means intended or anticipated by either party, and yet a most manifest result,) a substantial good will have been wrought.

It is to be hoped that the enterprise of those who have been so industriously seeking substitutes for vulcanite, is now crowned with success; a double good may thus be secured—escape from an annoying monopoly and the substitution of a better article. To those who do work vulcanite, however, it may be worth while to call attention to the methods of manipulating this material as introduced by Messrs. Stuck & Owens, some six years ago, the method bring briefly the hardening of the plate between sheets of tin. This method unquestionably has advantages over the ordinary style of vulcanizing, in bringing more clearly and in saving of time, and resulting in many cases in a better fit from the shrinkage of the metallic die on which the plate is hardened. It is worthy of the thoughtful experimentation of all who are interested in the subject.

The rise and progress of the celluloid base has been watched with absorbing interest by the profession at large, not so much as a means of escape from the vexatious Rubber question, as being the dawn of a hope of something better. This hope seems to have brightened into a substantial reality. The many experiments made by careful men have apparently favorably settled beyond all reasonable doubt some of the questions upon which the fate of this base seemed to hang. It does now seem demonstrated that with careful mangement it does not shrink or warp perceptibly, and the question of its durability also seems in a fair way of being favorably decided. All other things being equal it is esteemed by many as a better base than rubber, in that it is above the suspicion of mercural taint. But the dentist who expects to give it the slovenly manipulation he may bestow upon vulcanite with the expectation of a good result, will be disappointed, for the carelessness that may give a passable result in the one, will most probably result in the failure of the other. And in this feature of greater difficulty of manipulation, your committee see substantial merits.

Of the varieties of this collodion base, but two are prominently before the public, the so-called Celluloid and the Rose Pearl of Dr. McClelland. With the first it is suggested that when practicable, a metallic die or model will give more satisfactory results, avoiding the damage of breaking the plaster model. With the Rose Pearl much greater difficulties are present, owing to the hardness of the material, but the results are apparently excellent, plates moulded for months showing no signs of warping or shrinking. Some carefully conducted tests compel your committee to the belief that with careful manipulation excellent results may be derived from either of these varieties of the collodion base. It is hardly fair however to decide upon the merits of such a thing, upon the issue of a single trial, as some seem to have done, however confident we may be of our skill. But your committee would earnestly urge upon the members of this association a patient trial of one or both of these bases, not resting satisfied with a single failure.

---

